# Acetabular Central Fracture Dislocation after Generalized Seizure during Lumbar Myelography with Iohexol

**DOI:** 10.1155/2013/190917

**Published:** 2013-04-02

**Authors:** Kyung-Soon Park, Jae-Young Moon, Chang-Seon Oh, Taek-Rim Yoon

**Affiliations:** Center for Joint Disease, Chonnam National University Hwasun Hospital, 322 Seoyang-ro, Hwasun-eup, Hwasun-gun, Jeonnam 519-809, Republic of Korea

## Abstract

Fracture is a less common complication in seizure patients, and fractures as a consequence of convulsive seizures without direct trauma occur in 0.3% of cases. Acetabular fractures after convulsions are even more rare, and only a few cases of acetabular fracture dislocations, purely caused by convulsive activity, have been reported. Therefore, we report a case of unilateral acetabular central fracture dislocation after a seizure episode, with relevant literature review. The seizure attack occurred after contrast media (Iohexol) injection for checking the myelography.

## 1. Introduction

Seizure patients represent only 0.4% of emergency departmental visits, and 14% of these patients have accompanying injuries [1]. Fracture is not a common complication in seizure patients. Fractures as a consequence of convulsive seizures without direct trauma occur in only 0.3% of seizure cases [2]. Furthermore, fractures are directly related to trauma in 50% of those with a seizure-related fracture and only in 25% fractures are a consequence of seizure alone. Some of the more common locations for this type of trauma include the skull, proximal humerus, nasal bones, and clavicle [3]. Acetabular fractures after convulsions are extremely rare, and only a few cases of acetabular fracture dislocations, caused purely by convulsive activity, have been described in the literature. Here, we report a case of unilateral acetabular central fracture dislocation after a seizure episode without direct trauma and include a relevant review of the literature. The seizure attack in our patient occurred after contrast media (Iohexol) injection prior to myelography. Informed consent was taken prior to publication from the patient and her family. 

## 2. Case Report

A 73-year-old woman was referred to our hospital due to a left acetabular fracture. She had undergone L4-5 fusion for spinal stenosis 5 years ago, and after surgery she had persistent radiating pain. For pain control, a check lumbar myelography and spinal cord stimulator insertion was planned 4 months ago.

**Figure 1:**
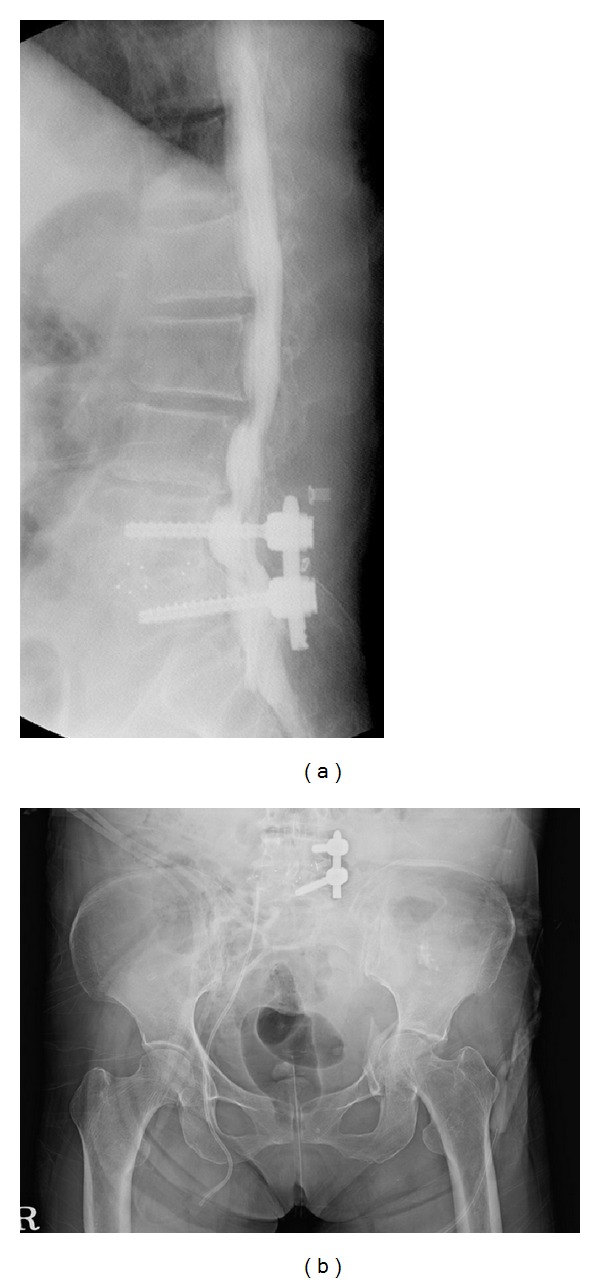
(a) Myelography taken after emergency care showing instrument on L4-5. (b) Radiograph taken 2 days after seizure episode showing left acetabular fracture with medial displacement of femoral head.

But she had a grand mal seizure when the myelography was being performed. The seizure developed at the moment of contrast media injection of about 7 mL of the total 10 mL (Figure 1(a)). The contrast media used was Iohexol (IO-Brix, Taejoon Pharm, Seoul, Korea). Emergency management was performed. During seizure attack, there was no fall or trauma. She had no history of a previous seizure.

**Figure 2:**
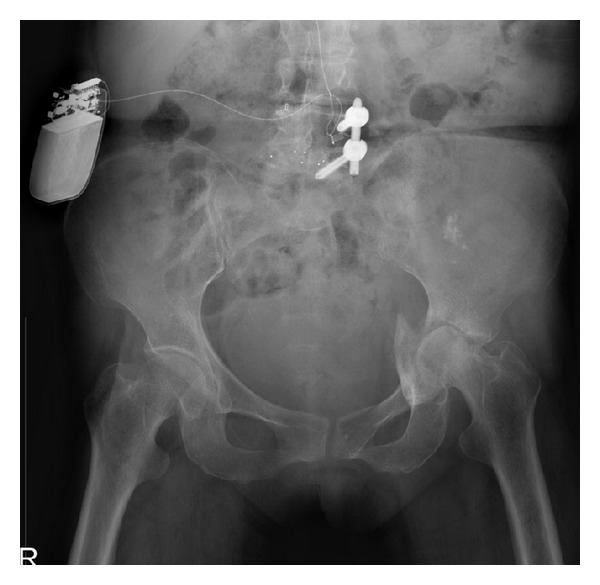
Radiograph taken 4 months after seizure episode showing left hip joint-space narrowing with femoral head destruction.

On regaining consciousness, she began to complain of increasing pain in the left hip, and 2 days after injury, plain radiographs were taken of the hip. The radiographs revealed an acetabular fracture with medial displacement of the left femoral head (Figure 1(b)). After discussion with the patient and her family, it was decided to treat the fracture nonoperatively at another hospital. Three days after the seizure, a spinal cord stimulator was inserted. During hospitalization, femoral skeletal traction of 9 kg was applied for 12 weeks to reposition the fracture dislocation. She was then discharged. However, the pain did not subside, and finally she was referred to our clinic, where plain hip radiographs showed disrupted congruity of the left hip and joint-space narrowing (Figure 2). The bone mineral-density scan done in our hospital revealed severe osteoporosis with T-score of −3.6 at L1–3 and −2.4 at proximal femur. Endocrine reference to diagnose secondary cause of osteoporosis was taken, but after complete evaluation, no cause was found and a diagnosis of postmenopausal osteoporosis was made. 

**Figure 3:**
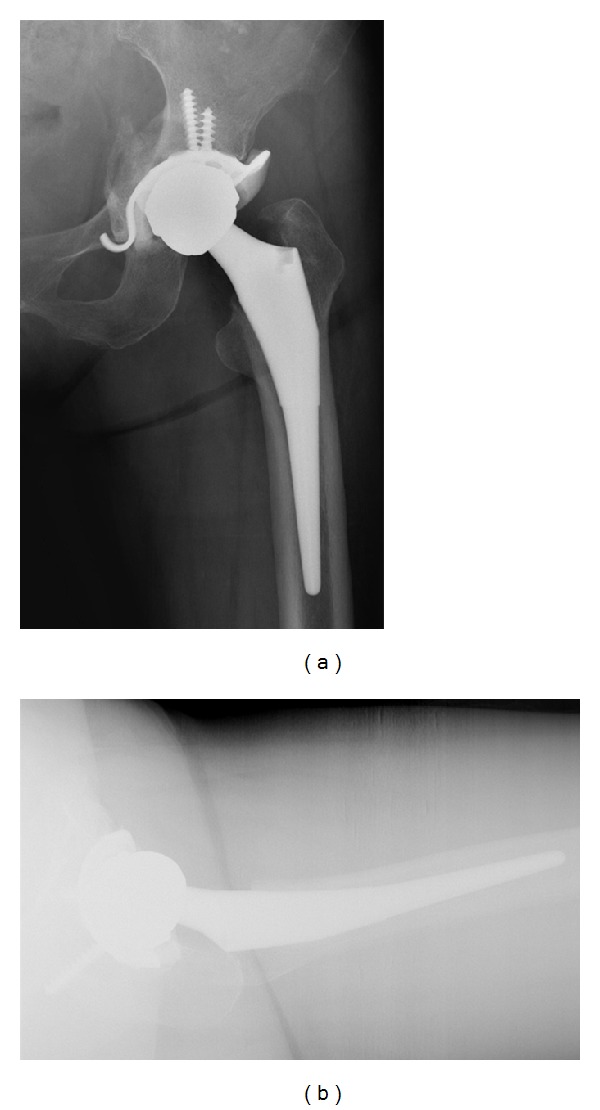
Radiographs taken 1 year after total hip arthroplasty showing well-fixed cementless femoral stem and consolidated medial acetabular bone graft with well-fixed acetabular cup ((a); (b)).

In view of the pain, joint-space narrowing, and femoral head destruction, we decided to perform total hip arthroplasty. The bone defect, caused by a previous fracture at the acetabulum, was filled with an autologous bone graft from the femoral head. To reconstruct the acetabulum, we used an acetabular roof-reinforcement ring with a hook (Ganz cup; Zimmer, Warsaw, IN, formerly Centerpulse, Winterthur, Switzerland) and a cementless tapered stem to reconstruct the femoral side (M/L taper; Zimmer, Warsaw, IN). After surgery, the patient was maintained non-weight-bearing for 6 weeks and then allowed partial weight-bearing. Full weight-bearing ambulation was allowed 3 months postoperatively. Till date, she had been followed for 1 year and there was no pain or limping on her left hip (Figure 3). Also, no other event of seizure attack was reported. 

## 3. Discussion

Fractures are a less common complication in seizure patients but have been reported to occur in 0.3 to 2.4% of patients who experience seizures [2, 3]. Of those diagnosed with a fracture, 50% had a fracture directly related to trauma, whereas only 25% had a fracture due to seizure activity alone, and in the other 25% fractures were undetermined [3]. One report on the same subject found that the proximal humerus was the most common fracture site in the atraumatic cases, and that the skull, nasal bones, and clavicle were the most common sites in the traumatic cases [2].

Fractures of the pelvis involving the floor of the acetabulum usually result from high-energy external trauma, such as a motor vehicle accident or a fall from height with direct impact. In patients with seizure disorders, the occurrence of acetabular fracture after seizure without accompanying direct trauma is extremely rare. One of the first reports issued on acetabular fracture resulting from seizure was published in 1944 by Haines, whose patient was on anticonvulsive therapy [4]. 

Contrast media is another rare cause of acetabular fractures after seizure, as described by Eastwood et al. [5]. Although contrast media have been used routinely for excretory urography, CT, and angiography for the past 30 years and are relatively safe, adverse reactions can be expected; the overall rate ranges from 5% to 8% [6]. The incidence of seizure induced by myelography in nonepileptic patients is very low, ranging from 0 to 0.847% [7]. Furthermore, seizure risk with nonionic, water-soluble contrast media, such as Iohexol is still lower and only few episodes of seizure attacks on clinical use have been reported [8, 9].

Several factors could increase the fracture risk in seizure patients. Uncontrolled massive muscle contraction being one reason, and if they occur around the hip, in a craniomedial direction, especially in an osteoporotic bone, the force generated could be strong enough to lead to an acetabular fracture [10]. 

This case highlights the importance of further evaluation in elderly patients that have experienced an epileptic attack after complete recovery from the seizure episode. Furthermore, evaluations of extremity pain, deformity, ecchymosis, and crepitus should aid the identification of bone injury after a seizure and always be followed by radiographs of the affected area. The current case provides an example of a rare fracture pattern caused by a seizure attack which occurred after Iohexol injection for check myelography, and alerts physicians of the diagnostic implications of this kind of fracture. Hence, the possibility of an acetabular fracture-dislocation should be kept in mind when a patient complains of hip pain or cannot walk after a seizure, especially a very osteoporotic elderly patient. 

## References

[1] Buck D, Baker GA, Jacoby A, Smith DF, Chadwick DW (1997). Patients’ experiences of injury as a result of epilepsy. *Epilepsia*.

[2] Finelli PF, Cardi JK (1989). Seizure as a cause of fracture. *Neurology*.

[3] Friedberg R, Buras J (2005). Bilateral acetabular fractures associated with a seizure: a case report. *Annals of Emergency Medicine*.

[4] Haines HH (1963). An unusual complication of convulsive therapy. *The Psychiatric Quarterly*.

[5] Eastwood JB, Parker B, Reid BR (1978). Bilateral central fracture-dislocation of hips after myelography with meglumine iocarmate (Dimer X). *The British Medical Journal*.

[6] Foote GA, Koelmeyer TD, Eyre KED, Astley TM (1998). Complications of epilepsy and a rupture pyonephrosis: radiology to the rescue in the brooks murder case. *Australasian Radiology*.

[7] Hughes CA, O’Briain DS (2000). Sudden death from pelvic hemorrhage after bilateral central fracture dislocations of the hip due to an epileptic seizure. *The American Journal of Forensic Medicine and Pathology*.

[8] Tahta K, Ozgen M, Berker M, Ciger A (1993). Status epilepticus following iohexol myelography. *Neuroradiology*.

[9] Song KJ, Lee KB (2004). Generalized tonic-clonic seizure following myelography with Iohexol (omnipaque): a case report. *Journal of the Korean Orthopaedic Association*.

[10] Sikkink CJJM, van der Tol A (2000). Unilateral transverse acetabular fracture with medial displacement of the femoral head after an epileptic seizure. *Journal of Trauma*.

